# Visualising the molecular alteration of the calcite (104) – water interface by sodium nitrate

**DOI:** 10.1038/srep21576

**Published:** 2016-02-15

**Authors:** Sascha Hofmann, Kislon Voïtchovsky, Peter Spijker, Moritz Schmidt, Thorsten Stumpf

**Affiliations:** 1 Institute for Nuclear Waste Disposal, Karlsruhe Institute of Technology, Hermann-von-Helmholtz-Platz 1, 76344 Eggenstein-Leopoldshafen, Germany; 2 Physics Department, Durham University, South Road, Durham, DH1 3LE, UK; 3 Department of Applied Physics, COMP Centre of Excellence, Aalto University, P.O. Box 11100, FI-00076 Helsinki, Finland; 4 Helmholtz Zentrum Dresden Rossendorf (HZDR), Institute of Resource Ecology, P.O. Box 10119, 01314 Dresden, Germany

## Abstract

The reactivity of calcite, one of the most abundant minerals in the earth’s crust, is determined by the molecular details of its interface with the contacting solution. Recently, it has been found that trace concentrations of NaNO_3_ severely affect calcite’s (104) surface and its reactivity. Here we combine molecular dynamics (MD) simulations, X-ray reflectivity (XR) and *in situ* atomic force microscopy (AFM) to probe the calcite (104) – water interface in the presence of NaNO_3_. Simulations reveal density profiles of different ions near calcite’s surface, with NO_3_^−^ able to reach closer to the surface than CO_3_^2−^ and in higher concentrations. Reflectivity measurements show a structural destabilisation of the (104) surfaces’ topmost atomic layers in NaNO_3_ bearing solution, with distorted rotation angles of the carbonate groups and substantial displacement of the lattice ions. Nanoscale AFM results confirm the alteration of crystallographic characteristics, and the ability of dissolved NaNO_3_ to modify the structure of interfacial water was observed by AFM force spectroscopy. Our experiments and simulations consistently evidence a dramatic deterioration of the crystals’ surface, with potentially important implications for geological and industrial processes.

Calcite is among the most common minerals in the earth’s crust^1^. It plays a major role in many geological^2^ and biological^3^,^4^ systems and is central in the preservation of the biosphere through its regulation of oceans’ acidity^5^. It is also central to several industrial processes such as cement manufacturing 6, in the polymer industry^7^, wastewater treatment^8^, nuclear waste storage^9^, and in the petroleum industry^10^. Calcite is part of the carbon cycle, and it is formed via precipitation from solvated calcium and carbonate ions as a result of supersaturation or as a biomineralisation product^11^.

The surface of calcite can be highly dynamic and evolve rapidly depending on its environment. The (104) surface plane (inset in Fig. 1) is the most stable thermodynamically and hence the most common in nature. In solution, equilibrium exists between the dissolution and re-precipitation of the crystal whose recrystallisation rate strongly depends on pH, CO_2_ partial pressure, and the presence of ions in the aqueous phase. The high reactivity of the mineral is confirmed by the large amounts of impurities, i.e. guest cations and anions, naturally occurring in calcite^12^,^13^,^14^. In the case of metal ions, recent studies^2^,^15^ have shown that the incorporation process is largely governed by the molecular organisation and dynamics of water in contact with the mineral’s surface, highlighting the importance of surface defects. This may not be true for larger organic molecules such as alcohols that can also interact with the surface of calcite in solution^16^,^17^,^18^,^19^,^20^.

The natural abundance of calcite, its high surface reactivity and its ability to incorporate foreign ions and molecules could offer viable new opportunities for the removal of heavy metals and pollutants^12^,^21^,^22^,^23^,^24^,^25^. However, in order to exploit this potential, it is crucial to understand the molecular structure and properties of the region where these processes take place: the interface between calcite’s surface and the solution containing the solute of interest. This interface comprises both the exposed surface of the crystal and the liquid molecules in immediate contact with it. The interfacial reactivity depends on the speciation close to the surface, and hence in the adsorption of contaminants, electrolytes and other sorbates originating from the bulk solution. Calcite’s interface exhibits remarkably well-structured water layers away from singularities such as step-edges^15^,^17^,^19^. This ‘interfacial liquid’ can prevent direct adsorption of single alkali ions^15^, emphasizing the need for a better understanding of the mechanisms allowing adsorption and incorporation of foreign molecules and ions.

Recent results^18^ have shown that even trace concentrations of sodium nitrate in aqueous solution, down to μM, can severely modify the calcite surface. In the presence of nitrate, an amorphous, comparatively soft layer develops on top of the (104) surface plane, coinciding with partial dissolution of the calcite matrix. Dramatic changes in the sorption behaviour of Ln^III^ due to this nitrate-induced effect were found by site-selective laser fluorescence spectroscopy of Eu^III^. Despite these findings, a clear molecular-level picture of nitrate’s effect on the interface is still lacking and is the object of the present study.

Here we investigate the interface between calcite (104) and aqueous solutions containing sodium nitrate. Nitrate is among the most common soil and water contaminants due to waste treatment processes, fertilizers and pore water evolution during degradation of cement^26^. The amount of dissolved nitrate in ground and pore waters highly depends on local conditions, and on the origin of nitrogen input. Concentrations of up to 10^−3^ M have been reported in ground waters^27^,^28^. In order to derive a complete molecular-level understanding of the calcite (104) – water interface in the presence of nitrate, we combined results from molecular dynamics (MD) simulations, X-ray reflectivity (XR), high-resolution amplitude-modulated atomic force microscopy (AM-AFM) and AFM spectroscopy. Our goal is to determine the details of the effect of NO_3_^−^ on the surface of calcite (104) at the sub-nm to sub-Å scale.

## Results

### MD simulations of the calcite/water interface in the presence of NaNO_3_/CaCO_3_

**Figure 1:**
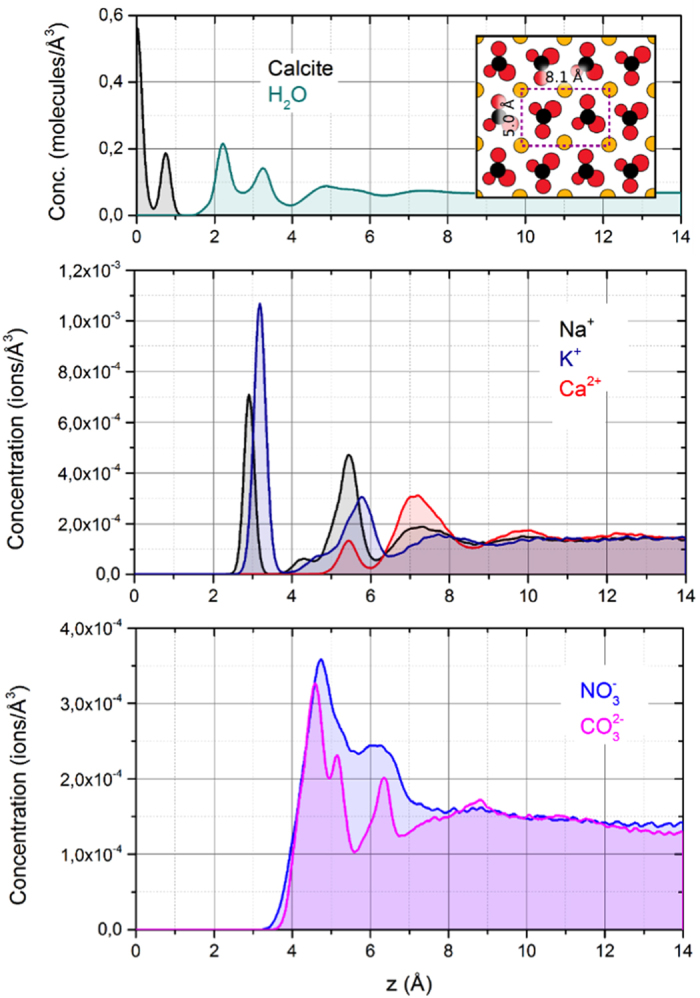
The simulated distribution of water and various dissolved ionic species on the calcite (104) surface. In the top panel, only the calcite and water (oxygen only) densities are shown, indicating the different hydration layers. The inset shows the crystal structure of the exposed surface as obtained from the simulations (calcium atoms appear yellow, carbon atoms black and oxygen atoms red). The middle and bottom panel show the cationic and anionic densities, respectively. The water density profiles are not appreciably affected by the presence of the ions.

We performed MD simulations of a system comprising a 40 Å thick slab of calcite (104) covered by about 100 Å of water in which 100 ion pairs were dissolved. After an initial equilibration period, acquisition was obtained over 5 ns runs (see methods for details). One of the strengths of MD simulations is the ability to access the positions of each atom throughout the entire duration of the simulations. It is, thus, straightforward to compute the densities of each atomic or molecular species perpendicular to the calcite surface, as seen in Fig. 1. In this figure the densities for water and for the atoms in the topmost layer of calcite are shown, perpendicular to the (104) surface. The occurrence of at least three hydration layers is visible from the density of the water molecules (cyan curve, only water oxygen atoms). This hydration structure is in good agreement with simulations by Gale *et al.* and also qualitatively similar to previous experimental findings^2^,^29^.

In the middle and bottom panel the ion densities for cationic (Na^+^, K^+^, Ca^2+^) and anionic (NO_3_^−^ CO_3_^2−^) species are shown. As previously observed^15^, only the small Na^+^ cations are able to penetrate the strongly bound hydration layers and can be found between the first and second adsorbed H_2_O layer at a height of 2.9 Å, but also at a broader region around 5.5 Å. K^+^ ions are located farther from the surface, at 3.2 Å. This observation coincides with the larger ionic radius of K^+^ ions with 1.38 Å in comparison to r_i_ = 1.02 Å for Na^+^. Both cations adsorb above the carbonate groups of the surface within the second hydration layer. The divalent Ca^2+^ ion, with its larger and stronger bound hydration shell, is not able to penetrate the adsorbed water layers of the (104) surface, concentrating mainly above the hydrated surface due to its high dehydration penalty^30^.

The multi-atomic nitrate and carbonate anions cannot reach past the second hydration layer, but are able to pass the third layer and aggregate directly on top of the second adsorbed water layer. Integration of the respective ion densities (integration window 3–10 Å) reveals that nitrate is 25% more concentrated than carbonate at the interface. This aggregation clearly shows the high affinity of nitrate towards the calcite surface, even exceeding that of carbonate- a singularity that could explain the unique effect of NaNO_3_ upon calcite. The affinity of nitrate towards the calcite surface can eventually lead to some substitution of NO_3_^−^ for CO_3_^2−^. Such would also affect the local hydration structure due to nitrate’s lower charge.

**Figure 2:**
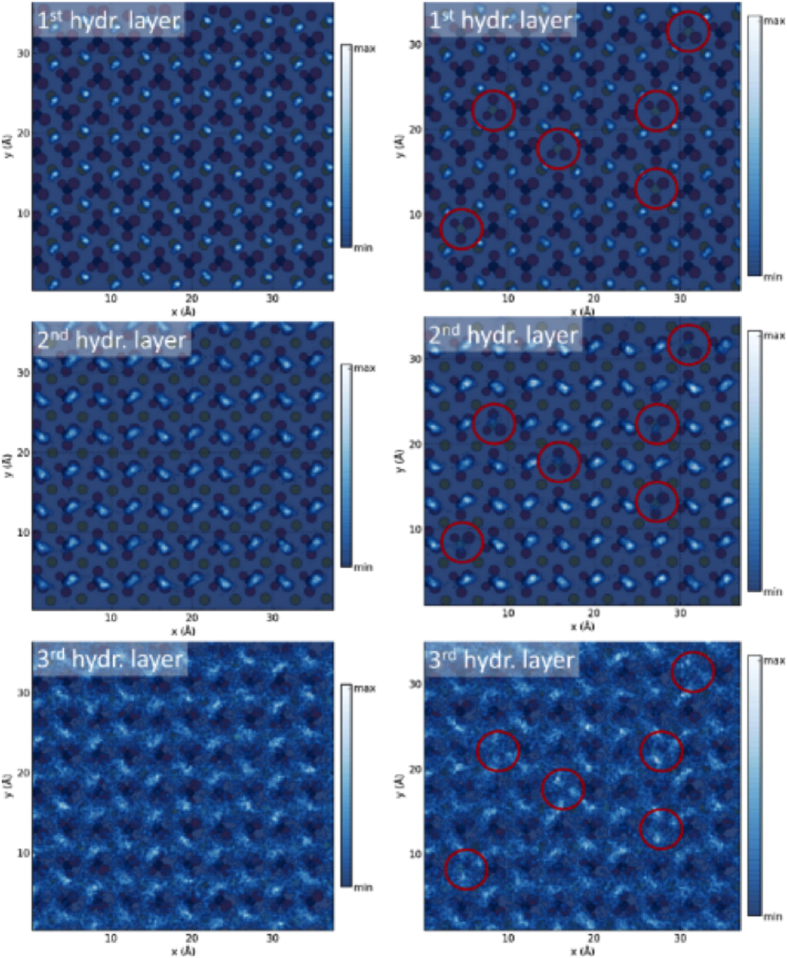
The lateral water oxygen density taken in planes parallel to the surface are shown for each of the three hydration layers (top to bottom). The panels on the right side show a calcite surface plane with six nitrate anions substituting for carbonate (red circles indicate their crystal location). The underlying crystal structure is visible in the background.

Figure 2 displays the lateral density of water’s oxygen atoms in planes taken parallel to the surface, at the location coinciding with each of the three different hydration layers. From these density profiles the characteristic zig-zag pattern for calcite (templated by the alternating protruding oxygen atoms of the carbonate groups) reported in previous AFM experiments^15^,^18^ can be easily identified. Based on the assumption that the high affinity of NO_3_^−^ towards the calcite surface will eventually lead to a substitution of CO_3_^2−^ by NO_3_^−^, a second simulation was run where six surface carbonate groups within the surface plane were randomly replaced by nitrate ions. Their position is marked by red circles in the right half of Fig. 2. The first hydration layer, being located above the calcium ions, is not affected by this incorporation. The second layer, however, exhibits definite changes with reduced water density on the surface, leaving the incorporation sites less hydrated within this layer. The overall structure of the liquid is disrupted at these sites which also influences the third hydration layer. There, areas above the nitrate groups show increased water density by a shift of water away from the surface.

### Microscopic insights of the surface modifications from AFM

**Figure 3:**
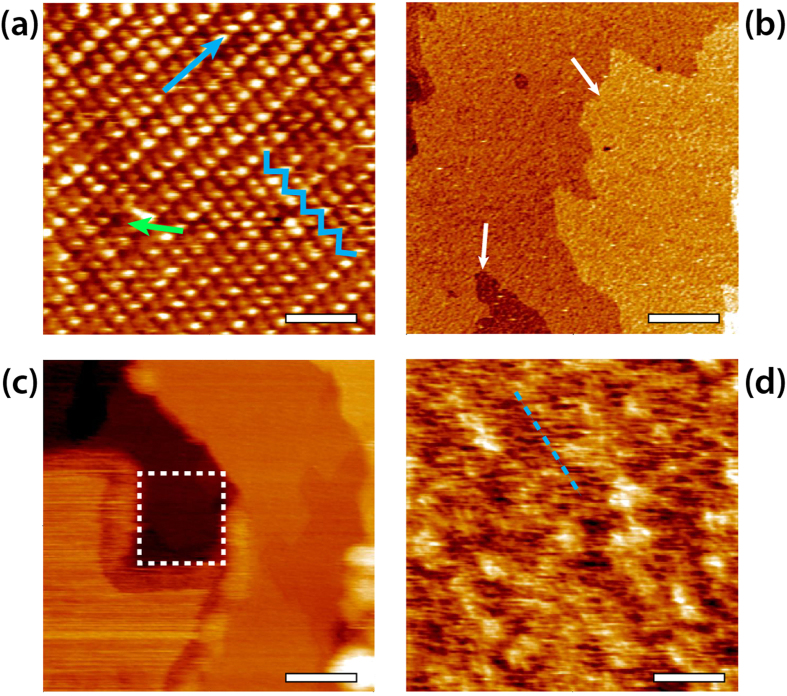
High-resolution imaging of calcite (104) in CSS with the oxygen atoms of the carbonates clearly visible as protrusions along the [010] direction (blue arrow) (**a**). The characteristic zigzag pattern is highlighted in the same colour. Occasional point defects are visible (green arrow). The presence of NaNO_3_ dramatically modifies the surface (**b**–**d**) with frayed edges (white arrows in b) and a soft surface layer that can be broken by the AFM tip using harsh imaging conditions (dotted square in c), revealing a crystal surface similar to (**a**) underneath. Over flat regions of the NaNO_3_ - modified crystal, it is possible to observe small clusters and hints of directionality (blue dotted line), suggesting epitaxial effects from the underlying crystal. All images are topographic images. The scale bars are 2 nm (**a**), 200 nm (**b**) and 20 nm (**c**–**d**). The images colour scale represents a height difference of 0.35 nm (**a**), 2 nm (**b**), 5 nm (**c**) and 0.8 nm (**d**).

In order to gain local, nanoscale insights into the effect of sodium nitrate, we compared amplitude-modulation AFM (AM-AFM) images of calcite obtained in a calcium saturated solution (CSS) and in a CSS containing 10 mM NaNO_3_. AFM images indicate that nitrate dramatically affects the surface of calcite, creating an amorphous surface modification (Fig. 3).

The effect of nitrate is particularly visible at steps^18^, suggesting that possible incorporation of nitrate into the calcite surface is likely to occur preferentially at singularities such as steps where the hydration layers are disrupted, thus easing the close approach of nitrate to the crystal surface. Several nitrate-induced aggregates can also be seen in the middle of the crystal surface (Fig. 3d). Although these aggregates appear to loosely orient along preferential directions, no atomic details could be resolved by AFM, suggesting that they are indeed amorphous. The observation of single adsorbed ions on these layers was not possible, unlike on the (104) planes of the crystal^15^.

The hydration enthalpy of the dissolved ions (−365 kJ/mol for Na^+^) is comparable to that of nitrate (−300 kJ/mol), while Ca^2+^ and CO_3_^2−^ exhibit a higher value (−1505 and −1315 kJ/mol, respectively)^30^. The approaching ions must either displace the hydration layers naturally present at the (104) surface of calcite in solution or lose their hydration shell in order to reach the mineral. This, however, is only valid for the (104) surface. The adsorbed water structure at step edges and kinks is less rigid presumably allowing for a close approach of ions to the crystal surface^31^,^32^. Consistently, AFM and MD simulations did not identify metal ions directly adsorbed onto calcite’s surface, but rather located at least one water layer away from the surface. Here, since stable point defects can be identified in the surface, we decided to explore experimentally the structure of interfacial water layers at the surface of calcite (104) in the presence and in the absence of sodium nitrate.

### Force spectroscopy measurements of the interfacial liquid

**Figure 4:**
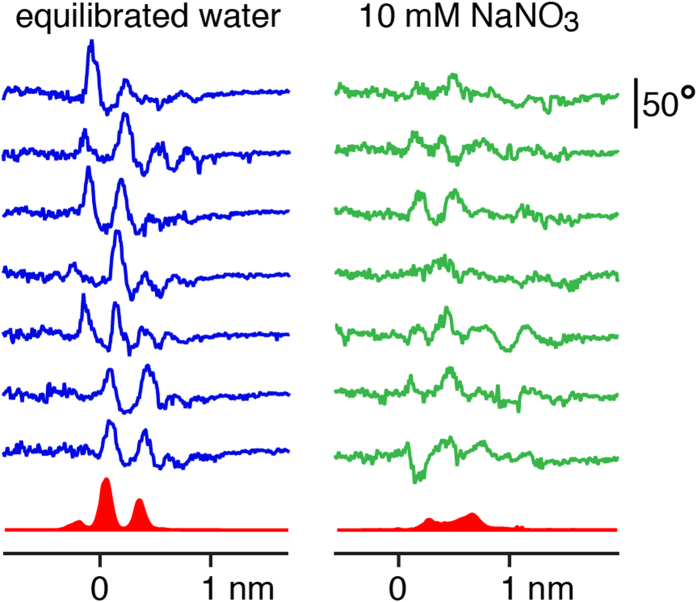
Representative force spectroscopy curves acquired by AFM at the interface between calcite (104) (left) and CSS, and containing 10 mM NaNO_3_ (right). The curves have been offset vertically for visibility and the averaged behaviour over more than 50 curves is shown in solid red at the bottom in each case. The curves show the oscillatory behaviour in the phase of oscillation experienced by the vibrating AFM tip as it approaches the calcite surface in solution. It is therefore expressed in degrees. The tip approach is from the right in both cases and the zero is defined (arbitrarily) as the position where the average cantilever deflection becomes positive. The analysis procedure used to align the curves and deduce the average position of the maxima is given in supplemental information (Figs S1 & S2). The amplitude of the statistical averages (red) is in arbitrary units, but directly comparable between both solutions (see Fig. S2 for more details).

In order to probe the effect of sodium nitrate on the interfacial hydration layers, we reduced the tip vibration amplitude to 1.2 Å, less than the thickness of a single water layer. Although not ideal for imaging weakly adsorbed ions^15^,^33^, this approach is more effective when investigating water density oscillations at interfaces. The same tip was used to compare the interface between calcite (104) and (i) CSS, (ii) a CSS containing 1 mM NaNO_3_ and (iii) a CSS containing 10 mM NaNO_3_. Representative results for (i) and (iii) are presented in Fig. 4. The results acquired in (ii) showed an intermediate behaviour making any averaging ambiguous (see supplementary Figs S1 and S2).

As expected from MD simulations and reflectivity measurements, the oscillatory behaviour is pronounced in water with typical curves exhibiting well-defined maxima. Although the curves were acquired randomly over calcite’s 104 surface, their reproducibility suggest a behaviour dominated by the most prominent oxygen atoms of the surface. However, the first hydration layer of calcite’s surface remains un-probed here due to the relatively soft cantilever used (see Methods section), which makes it impossible for the AFM to fully remove the water between the tip apex and the calcite surface^34^. Consequently, the signature of the first hydration layer around 2.3 Å from the surface is not visible in the spectroscopic curves. It should also be noted that the position of the zero on the horizontal axis represents the position where the vibrating cantilever starts to experience a continuous positive deflection when pressing against the crystal. This position serves as a reference to align the curves, but it is an arbitrary reference that does not necessarily coincide with the absolute position of the crystal’s surface. Only relative distances (e.g., between adjacent maxima) should therefore be considered. More than 50 curves were acquired over the sample and averaged to provide a statistically significant result (bottom of Fig. 4). The oscillatory pattern is clearly visible in the average and comprises information about both the tip and the sample’s hydration structures. The measured distances between the main adjacent maxima are 2.3 ± 0.3 Å and 2.9 ± 0.2 Å, respectively. Although these distances could be explained by local variation of the water density over the surface (the water density profile is not the same above calcium or carbonate), it seems more reasonable to ascribe the observation to the convolution effect between the hydration layers of both tip and surface^35^.

In contrast, measurements in the nitrate solution do not show a reproducible oscillatory behaviour. Many curves do not show any clear oscillation while others exhibit oscillations with a larger wavelength. A statistical analysis of >50 curves confirms the overall disappearance of the oscillatory pattern, with two broad maxima 3.8 ± 0.7 Å apart.

Comparing the results in CSS and in nitrate-CSS indicates that nitrate is able to interfere with the intrinsic hydration structure of calcite and penetrate the near-surface water layers. AFM is by definition a perturbative measurement and the influence of the tip on the measurement (e.g. forcing dissolved nitrate into the interfacial layers during the measurements) cannot be excluded^36^. Nonetheless, direct comparison between results obtained in both solutions unambiguously demonstrate that nitrate influences the organisation of water at the interface, down to the closest water layers. A more direct comparison between the oscillation curves and the interfacial structure is presented in the next section.

### X-ray reflectivity of the destabilised calcite/water interface

**Figure 5:**
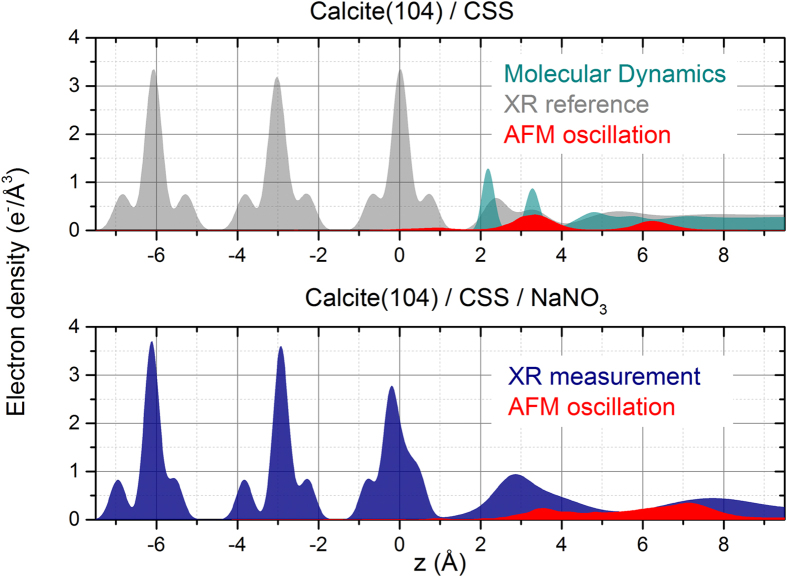
Top: Electron density plot along surface normal direction derived from CTR measurements of a calcite surface in contact with equilibrated solution from Fenter *et al.* (grey)^29^. The electron density of H_2_O in a calcite/water system computed directly from atomic positions from the MD simulations is plotted as dark cyan area. The AFM oscillation curve from the CSS system was added as red area for comparison of the different approaches. Bottom: Electron density profile of calcite in contact with 1 mM NaNO_3_ CSS for two weeks reaction time obtained from CTR data (blue). The oscillation curve obtained from 10 mM NaNO_3_ solution is added in red.

X-ray reflectivity measurements (reflectivity scans at a specular arrangement) were performed at an incident X-ray energy of 16.0 keV resulting in the shape of a crystal truncation rod (CTR). The total electron density calculated from the crystal truncation rod of the (104) surface in contact with NaNO_3_ is displayed in Fig. 5. The surface structure model includes contributions from the first six monolayers of the crystal, a layered water model and surface adsorbed species. For better comparison with the calcite/CSS reference system, a density profile calculated from Fenter *et al.*’s work^29^ is added in Fig. 5.

When in contact with nitrate, striking differences of the upmost three monolayers’ structure become apparent. The tilt and rotation angles θ and Φ of the carbonate groups, respectively, are strongly distorted from their ideal bulk positions and also in comparison to the surface-relaxed reference in contact with CSS. Along with this change, atomic positions of Ca^2+^ and CO_3_^2−^ (followed by the carbon atom) severely differ from reported values. As a result, the shape of the vertical electron density of the monolayers is modified, creating a smaller gap between the first and second layer of the crystal’s surface and bringing them closer to each other by ~0.3 Å. The interfacial liquid structure is also affected by the presence of NaNO_3_. The two adsorbed water layers, well documented in the literature, disappear leaving only one distinct peak at 3.4 Å distance from the crystal. This peak exhibits largely increased electron density of ~2.3 e^−^/Å^3^ which reduces to bulk level (0.33 e^−^/Å^3^) over 12 Å from the surface. The increased electron density of the interface is similar to that of a calcite monolayer (~2.5 e^−^/Å^3^) and cannot be caused by the structuring of H_2_O molecules alone. The most reasonable explanation to the changes observed in this region is therefore the presence of NaNO_3_. The electrolyte may trigger the agglomeration of ions to form amorphous, hydrated surface layers. This is in good agreement with earlier microscopic findings^18^, where such a layer on top of the calcite crystal surface could be observed. Qualitatively comparing these electron density profiles with the AFM force spectroscopy curves obtained in this study reveals similarities between the results obtained from both methods (Fig. 5). Some agreement between AFM oscillation peaks and locations of increased electron density can be found in both systems. Both oscillation peaks from the nitrate containing system are in accordance with the sorption peaks found by XR. Their broad shape, being considerably less distinct compared to the nitrate-free system, is also reproduced consistently. In AFM the first oscillation peak of the nitrate containing system exhibits a lower amplitude than the second one, contrarily to the X-ray electron density. This could be attributed to the high concentration of ions in this adsorbed, amorphous film creating high electron densities in conjunction with a pronounced softness as shown by the lower force needed by the AFM tip to penetrate the layer.

## Discussion

In this study, we combined MD simulations, X-ray reflectivity and AFM to gain insights into the destabilising and modifying effect of sodium nitrate on the surface of calcite crystals. A direct comparison between the XR results and the electron density computed from the atomic positions obtained by MD should be done with caution since MD simulations cover only a reaction time of 5 ns while the XR sample was in contact with NaNO_3_ for 2 weeks. Similarly, the nitrate concentrations used for MD, XR and AFM experiments differ significantly to compensate for practical constraints, such as the small sampling time available in MD (see Methods). Nonetheless, the fact that Na^+^ and NO_3_^−^ ions adsorb in close proximity to the surface in significant concentrations within nanoseconds, suggests that these play a major part in the restructuring process of the calcite surface. The higher affinity of nitrate towards calcite could trigger incorporation of the guest ion into the surface replacing carbonate. Our simulations show that this behaviour leads to a disrupted hydration structure with water density shifted away from the surface and a changed water coverage at the height of the third water layer. The electrolyte-driven formation of a non-crystalline phase on the calcite surface could be confirmed, revealing molecular-level details about the interface with the surrounding liquid. The presence of NaNO_3_ therefore changes the structure of both the calcite surface and the interfacial liquid substantially.

The behaviour of nitrate contrasts with that of other monovalent ions which, despite their ability to come close to the crystal’s surface, are not known for any specific effects such as changing the morphology or reactivity of calcite significantly^15^,^37^. The observed alkali ion densities are consistent with X-ray surface diffraction measurements in RbCl solution where the Rb^+^ cation was also observed between the first and second hydration layer and further away around 5–6 Å. Additionally, X-ray results found Rb^+^ ions at the calcite interface^38^, a result not reproduced here. We attribute this to the relatively short time scales accessible to MD simulations (several nanoseconds), or possibly to the absence of surface defects such as vacancies, dopants and steps that are able to break the strongly bound water hydration layers and allow ions to reach the surface. This second hypothesis would also be consistent with previous studies, which confirmed that ions eventually reach the surface such as inner sphere complexes of Cm^3+^ and Eu^3+ ^39,40.

The electron density profile, obtained by XR measurements, showed a dramatic rearrangement of the calcite structure up to three monolayers into the crystal. This destabilisation coincides with the dissolution effects observed by AFM where, especially at steps and edges, it creates frayed structures. The partial dissolution and overall destabilization of the first calcite monolayers is supported by XR; it was shown that the upmost layers with respect to atomic positions and carbonate tilt/rotation angles exhibit relaxation effects exceeding those of the pure system significantly. Re-precipitation as an amorphous phase directly above the crystal surface was also observed at ~3.4 Å, under loss of the two distinct adsorbed water layers. Its electron density is nearly as high as that of a calcite monolayer, but exhibits no structural features at all. AFM force spectroscopy measurements of the interface are in agreement with these findings, showing significant differences between solutions with and without NaNO_3_. The hydration structure of the interface in the pure calcite/CSS system is well depicted by clear oscillations. However, the distances between averaged maxima exceed those measured by XR and predicted by MD by >1 Å, a discrepancy that could be ascribed to the hydration structure of the measuring tip. We note that the distance between the peaks coincides with that between the first and third hydration layer and could in principle be explained by the particular location where each curve was acquired (Fig. 1b). The main finding is the dramatic difference to the NaNO_3_ bearing solution where only very broad maxima were detected, in accordance with the XR data. Since the clear structuring of the liquid has been shown to disappear in presence of NaNO_3_, and a non-crystalline layer forms on top of the surface, the AFM force measurements are in good agreement with the XR results. The two oscillation peaks’ distance of 3.8 ± 0.7 Å also is within the range of the distance of the two major peaks in the electron density profile plot. Combining the non-structured shape of the nitrate-induced layer regarding electron density and its characteristics seen by AFM, we propose that this amorphous layer is formed by an agglomeration of solvated ions. The material initially dissolved from the solid would re-assemble above the surface^41^. Short-range order and structural characteristics are in good agreement with earlier findings^42^. The question arises, however, as to how the presence of the very soluble electrolyte NaNO_3_ can lead to such consequences. Regarding the instantaneous (i.e. within 5 ns) attraction of ions to the calcite surface, our MD simulations give new insight. Nitrate and carbonate ions are not able to penetrate the adsorbed water layers of a pristine (104) surface within this short time, reaching maximum density at approximately 5 Å from the surface. The presence of point defects in the crystal (Fig. 3a), in particular those away from step edges could, however, provide a pathway for nitrate to directly reach the surface of calcite and interact with it. In the absence of such defects, the particularly robust hydration structure naturally present at the (104) surface is unlikely to favour direct interactions since it has been shown to prevent single hydrated alkali ions from reaching the crystal^15^. Nitrate was found to be 25% more concentrated at the interface than CO_3_^2−^ exhibiting strong affinity to calcite. Alkali ions are able to approach the calcite surface better than calcium and become located between the first and second hydration layer. Comparing sodium and potassium nitrate (see supplemental information) makes clear that nitrate ions have to be mainly responsible for the surface alteration and differences exist in the process-supporting behaviour of both cations. Together, this implies high affinities of Na^+^ and NO_3_^−^ for the calcite surface. Considering the high interfacial NO_3_^−^ concentration, incorporation would induce a disruption of the local hydration structure which could act as a starting point for the surface modification. We suggest that step edges, kinks and point defects are favoured locations for this process to happen since their hydration structure is more disturbed than that of the (104) plane^31^,^32^.

Our study sheds light on the mechanism of the reaction of calcite with solutions containing the electrolyte NaNO_3_. The formation of an amorphous phase was observed consistently and confirmed by a combination of microscopic, X-ray reflectometric and computational approaches. This film covers the crystal surface forming a layer that prevents direct interaction of ions and molecules with the calcite surface, for example reducing the uptake of rare earth elements^18^.

## Methods

### Preparation of calcite crystals

For all AFM and XR experiments, natural calcite single crystals (Iceland spar, purchased from Ward’s Science, NY/USA) were used. The crystals were cleaved along the (104) plane using a razor blade and immersed into their respective reaction solutions within seconds. All solutions were equilibrated with calcite and atmospheric CO_2_ prior to experiments. The crystals were stored in the calcite equilibrated solution (CSS) for one hour before NaNO_3_ was added. X-ray reflectivity scanning took place after two weeks contact time. For AFM measurements, the freshly cleaved crystals were stored in calcite equilibrated solution with concentrations of 1 mM NaNO_3_, respectively, for one week in a centrifuge tube. Crystals were taken out of solution shortly before experiments and fixed on a metal sample holder with epoxy resin. During this procedure, the surface was constantly covered by the reaction solution to exclude unwanted effects by precipitation or drying.

NaNO_3_ and calcite powder (for solution equilibration) was purchased from Merck Suprapure and used without further purification. The different solutions were prepared by dissolving the desired amount of chemical in equilibrated calcite solution. The latter was prepared by immersing calcite powder in ultrapure water (18.2 MΩ EMD Millipore, MA/USA) and stirring regularly until full equilibration (typically >1 week).

### Molecular dynamics simulations

In a previous study we quantified the distance between different hydrated ions (NaCl, RbCl, and CaCl_2_) and the surface of calcite^15^. For the current work we used the same systems, but replaced the hydrated ion species by either NaNO_3_ or CaCO_3_. We included the latter to also study the effect of the ion species that naturally occur in the experiments simply by equilibrium dissolution and recrystallisation of calcite. We followed the same MD simulations protocol as previously published^15^. In short, 10 different setups were generated (each with all the atoms/molecules of all 100 ionic pairs placed at different random locations and with different initial velocities) in order to improve statistical accuracy. Based on the system dimensions of 105 × 64 × 137 Å^3^ (of which 40 Å in the z-direction is the calcite crystal), the ion concentration is approximately 250 mM. We note that this concentration is considerably higher than that used in the XR and AFM experiments. However, the relatively small size of the system and the short time probed (compared to experiments) make it necessary to work with these conditions if to obtain any meaningful statistics; an approach which has been validated in previous studies^15^,^43^,^44^. Despite these high concentrations, aggregation happens only on a limited basis. This is, however, no longer the case when mixtures involving ions present in the CSS are introduced (e.g., NaNO_3_ and CaCO_3_ at the same time). In such cases, aggregation far away from the surface is the predominant process taking place, making it impossible to objectively gauge any effect on the behaviour of the ions near the solid-liquid interface.

The production run of 5 ns at ambient conditions (310 K and 1 bar) is preceded by short minimisation (500 steps) and equilibration runs (20 ps) in order to minimize stress in the system introduced by the artificial placement of the ion pairs. As before, all simulations are carried out using the molecular dynamics code NAMD^45^ with the empirical force field CHARMM^46^ dictating all atom interactions. As the nitrate ion is not part of the standard distribution of the CHARMM force fields, its Van der Waals^47^ and charge^48^ parameters were obtained elsewhere and converted to the proper units and functional forms as used in the CHARMM force field. The simulations are run in parallel on a typical Linux commodity cluster and analysis is performed visually using VMD^49^ or numerically using the Python library MDAnalysis^50^. Far away from the surface, the ion concentration is approaching that of the initial bulk concentration (250 mM). Overall, the striking resemblance of the density profiles derived from MD and from diffraction data further away from the surface strengthens our current results.

### X-ray reflectivity

X-ray reflectivity (XR) measurements were carried out at GSECARS undulator beamline 13 IDC of the Advanced Photon Source, Argonne National Laboratory. XR measurements of calcite have been conducted similar to experiments described in detail elsewhere^29^,^51^,^52^. The calcite structure is defined by a vertical layer distance of 3.04 Å and a unit cell area of 20.2 Å^2^. The measurements were performed at a non-resonant, incident X-ray energy of 16.0 keV. Reflectivity scans at a specular arrangement result in the shape of a crystal truncation rod (CTR). The reflectivity signal *R(Q)* was measured as a function of the vertical momentum transfer *Q* with *|Q|* = [4*π*(2sin θ/2)]/λ. Experiments were performed *in situ* in a thin-film cell with an approximately 5 μm thick water film on the crystal surface which can be pumped in or out^53^. Analysis of the XR data is described in detail in the supporting information.

### Atomic Force Microscopy

AFM measurements were performed on a Cypher ES microscope (Asylum Research, Oxford Instruments, CA/USA) with the tip fully immersed into the solution. The AFM was equipped with photothermal excitation of the cantilever (blue drive) for better stability and the sample’s temperature was kept constant at 25 °C in a sealed, *in situ* cell. The imaging was performed in Amplitude Modulation mode (AM-AFM) driving the cantilever close to its resonance frequency. We used ArrowUHFAuD cantilevers (Nanoworld, Germany) with a nominal spring constant of ~2 N/m and resonance frequencies varying between 400–430 kHz in liquid. Each cantilever was calibrated using the thermal method^54^ and we used cantilevers from the same batch for all samples for better comparison.

### Imaging

Atomic-resolution imaging was carried out at scan rates ranging from 3 to 10 Hz and cantilever oscillation amplitudes between 0.5 and 1 nm (peak to peak). The amplitude is chosen to probe the whole solid-liquid interface during each oscillation cycle^34^,^55^. Lower resolution images were achieved at lower scanning rates and amplitudes between 1 and 2 nm. Data analysis was carried out using the free software WSxM v5.0 Develop 6.5^56^ in combination with Igor Pro 6.3.4.1 software (Wavemetrics, Lake Oswego, OR/USA). Images were line-corrected for tilt but not filtered.

### Force measurements

In order to probe the structure of the liquid at the surface of calcite, amplitude- and phase-distance spectroscopy curves were acquired at different locations but always away from steps. In contrast to imaging, smaller tip oscillation amplitudes were used (typically, 0.12 nm) so as to probe individual water layers. As the tip crosses the different water layers, an oscillatory damping behaviour can be identified in amplitude and phase with characteristic maxima (Fig. 5). Statistics were conducted on the average distance between the maxima in the presence and in the absence of nitrate. At least 50 curves were used in each case, all acquired with the same tip (first in equilibrated solution, then in nitrate-containing solution) so as to ensure comparability. The statistical analysis was conducted using automated routines programmed in Igor Pro and described in supplementary information.

## Additional Information

**How to cite this article**: Hofmann, S. *et al.* Visualising the molecular alteration of the calcite (104) – water interface by sodium nitrate. *Sci. Rep.* **6**, 21576; doi: 10.1038/srep21576 (2016).

## Supplementary Material

Supplementary Information
