# Supplementary material for: Visualising the molecular alteration of the calcite (104) – water interface by sodium nitrate
Source: Sci Rep. 2016 Feb 15;6:21576. doi: 10.1038/srep21576 (PMC4753505; doi:10.1038/srep21576)
Supplement: Supplementary Information [file srep21576-s1.pdf]

Supplementary information

Visualising the molecular alteration of the calcite (104) – water interface by sodium nitrate

Sascha Hofmann[1], Kislon Voitchovsky[2,*], Peter Spijker[3], Moritz Schmidt[4] & Thorsten Stumpf[4]

[1]Institute for Nuclear Waste Disposal, Karlsruhe Institute of Technology, 1Hermann-von-Helmholtz-Platz, Eggenstein-Leopoldshafen 76344, Germany. [2]Physics Department, Durham University, South Road, Durham, DH1 3LE. [3]Department of Applied Physics, COMP Centre of Excellence, Aalto University, P.O. Box 11100, FI-00076 Helsinki, Finland. [4]Helmholtz Zentrum Dresden Rossendorf (HZDR), Institute of Resource Ecology, P.O. Box 10119, 01314 Dresden, Germany
Correspondence should be addressed to: K.V. (email: kislon.voitchovsky@durham.ac.uk)

Table of contents

Analysis method of the AFM spectroscopy curves 2
All AFM spectroscopy curves used for the analysis of the interface 3
X-ray reflectivity (CTR data analysis)..5
Additional data and discussion 8
References 10

Analysis method of the AFM spectroscopy curves

All the spectroscopy curves were acquired with a same cantilever/tip, first on calcite in equilibrated water solution and immediately after on calcite equilibrated in a solution containing 10 mM $NaNO_3$. In each case at least 50 curves were acquired, with AM-deflection, amplitude and phase recorded simultaneously.
A typical spectroscopic set of curves is presented in Fig. S2 a-c for measurements in equilibrated water. During the tip vibration damping, a typical oscillatory behaviour can be seen in amplitude and in phase (Fig. S1 b-c). These oscillations are characteristic of the tip traversing structured layers of water at the surface of the sample[1, 2]. The oscillatory behaviour is particularly obvious in the phase curve, and phase curves were therefore used for the automated statistical analysis described below.

First, all the curves were aligned based on the AM-deflection curve, with the zero piezo extension arbitrarily defined as the position where the average tip deflection becomes larger than zero (Fig. S1 a). The average phase shift was then removed from each phase curve by subtracting a sigmoidal fit (Fig. S1 c) so as to leave only the oscillatory behaviour (Fig. S1 d). The analysis was then conducted only on the damping region (region between brackets in Fig. S1 d) to minimize computing calculations. The selected region of interest (Fig. S1 e) was then analysed by performing a Gaussian fit over a 0.25 nm interval, taken around each point of the curve analysed. The resulting amplitude (Fig. S1 f) and width (Fig. S1 g) of the Gaussian for each point exhibit characteristic plateaus at oscillatory maxima and minima, reflecting fits converging to the same result. The centre position of the Gaussian fits also exhibits plateau in the same regions (not shown). 'True' maxima were then identified imposing the following conditions: (i) fit amplitude positive and larger than 5 standard deviations of the curve average, and (ii) fit width positive but smaller than 0.25 nm. All the points not satisfying the above conditions were set to zero, effectively imposing a filtration process removing singular fits, minima and unclear maxima.
Subsequently, an integration curve was obtained by summing for each point the Gaussian curve obtained from the retained fit parameters, after filtration (Fig. S1 h). Given the fact that most points near a maximum converge to the same fit, the integration process effectively amplifies the importance of true maxima. The result is a curve of arbitrary amplitude only reflecting the true oscillatory maxima and with a zero baseline.
This procedure was repeated for each phase curve. The resulting maxima were then aligned and averaged, as visible in Fig. S2 c-d.

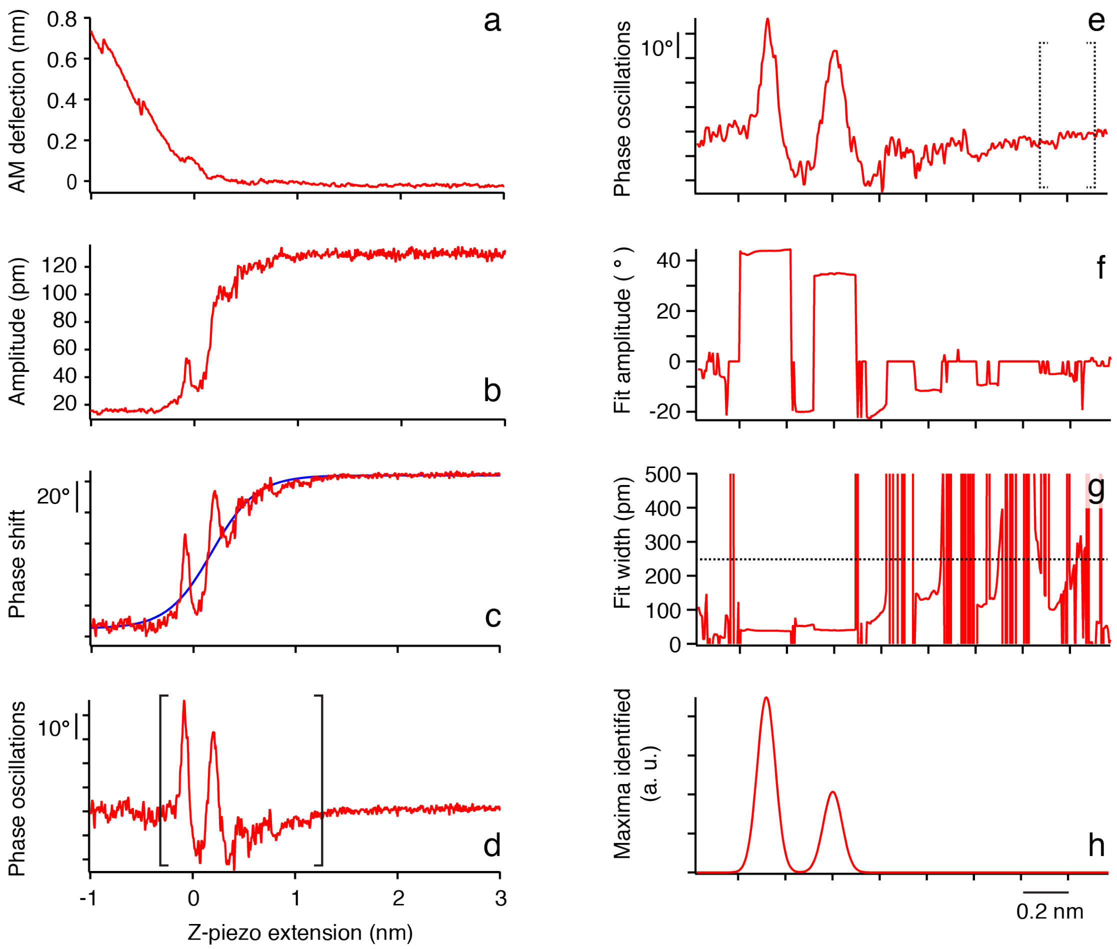

Fig. S 1. Example of the automated analysis procedure used to identify oscillatory maxima in the AFM spectroscopic curves. Each raw curve comprises AM-deflection (a), amplitude (b) and phase (c, red curve). The oscillatory behaviour, best visible in the phase, can be isolated by subtracting a sigmoidal fit (c, blue curve) from the raw data (d). The analysis focus on the damping region shown between the square brackets in (d) and reproduced in (e). The process consists for running a small interval along the curve (dotted brackets in e) and fitting each interval with a Gaussian curve. The resulting amplitude and width of the Gaussian obtained for the interval around each point are given in (f) and (g) respectively. A discrimination process of the fits allows retaining only the 'true' maxima (see text) which are emphasised by an integration process (h).

All AFM spectroscopy curves used for the analysis of the interface

Fig. 4 of the paper shows some representative spectroscopic phase curves after isolation of the oscillatory behaviour (subtraction of a sigmoidal fit, described in section 1). Hereafter, all the curves used for the statistical analysis are presented, after having undergone the same procedure.

The resulting maxima identified are also shown after alignment. Their averaging yields the curves presented in Fig. 4 of the paper.

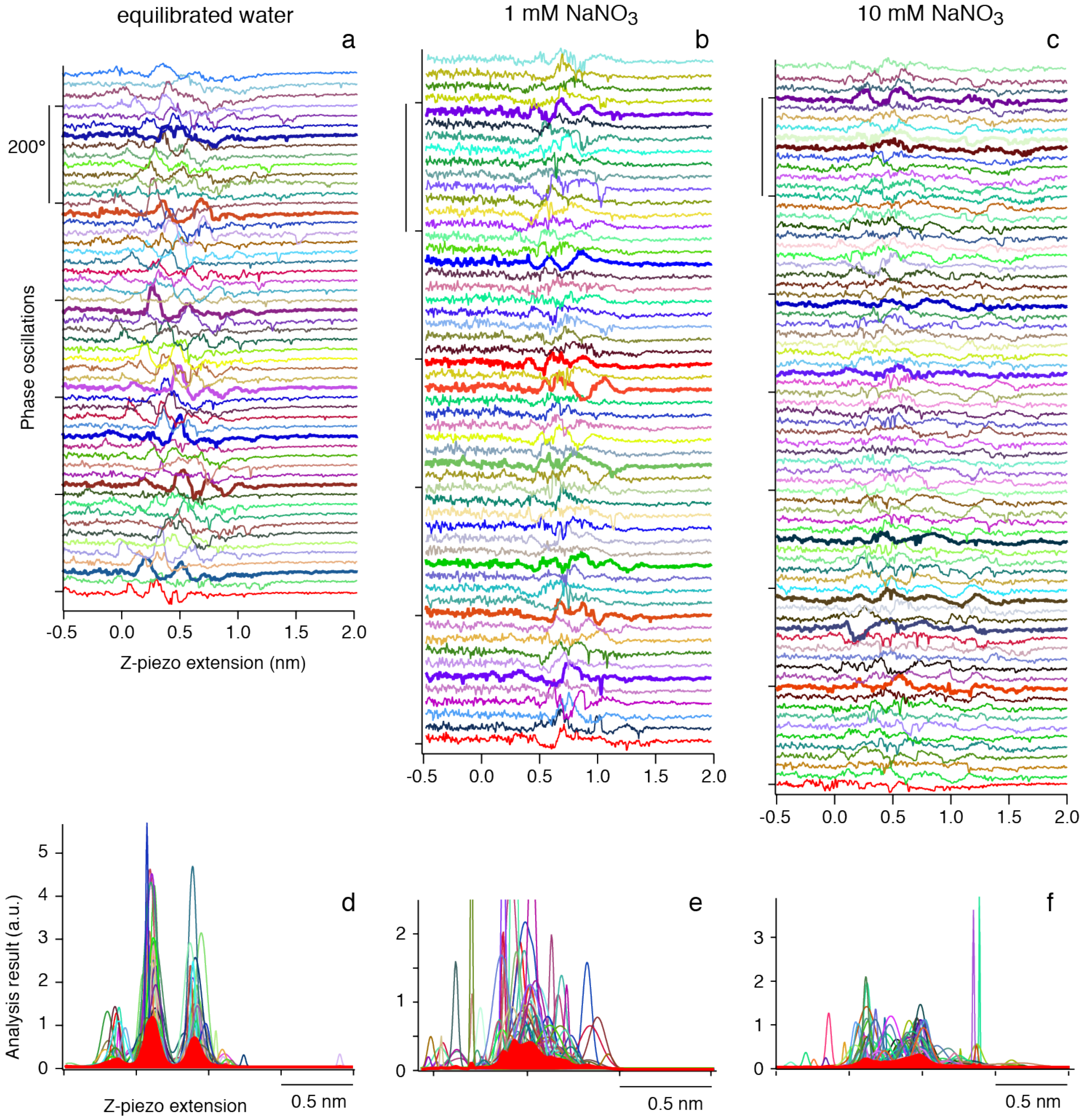

Fig. S 2 All phase curves used for the spectroscopy analysis. The damping-induced phase shift has been subtracted leaving only the oscillatory behaviour (a-c). All the curves have been vertically offset for better visibility, and aligned based on their respective AM-deflection (see section 1). After analysis, the maxima identified for each curve are aligned and averaged (d-f). The oscillatory behaviour is more marked in equilibrated water than in sodium nitrate, as visible by the larger amplitude of the identified maxima and their respective average (solid red). The influence of sodium nitrate appears progressively with concentration, with many curves in 1 mM $NaNO_3$ looking similar as in equilibrated solution. At 10 mM $NaNO_3$, all the curves are affected by the presence of nitrate.

X-ray reflectivity (CTR data analysis)

X-ray reflectivity measurements were conducted at the GeoSoilEnviroCARS 61 undulator beamline 13 IDC at the Advanced Photon Source, Argonne National Laboratory. The beam has a typical flux of ~ 1012 photons/s from a cryogenically-cooled double crystal Si (111) monochromator. The beam is collimated by a pair of 1 m-long Kirpatrick-Baez mirrors and the final beam profile of 0.05-0.25 mm (horizontal) by 0.5-1.0 mm (vertical) is defined by slits. We used a Dectris PILATUS 100 K 2D pixel array detector allowing the simultaneous recording of signal and background in one image.

Calcite single crystals were freshly cleaved prior to experiments and put into solution immediately. The solution was equilibrated with calcite powder ($CaCO_3$, Ultrapure, MERCK) and air beforehand to exclude dissolution effects. The crystals were stored in this solution, containing 1 mM $NaNO_3$, for two weeks. Experiments were performed in situ in a thin-film cell with a 1 – 5 µm thick solution film on the crystal surface which can be exchanged by a peristaltic pump. Kapton film (thickness = 7.5 µm) was used to prevent drying of the solution film. We measured the specular crystal truncation rod (CTR) using an incident energy of 16 keV. The measured (00L) rod connects Bragg peaks at L = 2, 4 and 6 in reciprocal space. The reflectivity signal R(Q) was measured as a function of the vertical momentum transfer Q,

$$|Q| = \frac{4\pi}{\lambda}\sin\left(\frac{2\theta}{2}\right).$$

R(Q) is a direct result of the interfacial structure and its structure factor which is described by the atoms at the interface:

$$R(Q) = \left(\frac{4\pi r_e}{QA_{UC}}\right)^2 |F_{UC}\cdot F_{CTR} + F_{int} + F_W|^2$$

r_e is the classical electron radius (r_e = 2.818 x 10^{-5} Å). The relevant atoms of the interface are divided into subsets: The calcite unit cell (F_{UC}), CTR (F_{CTR}), interface (F_{int}) and bulk water (F_W). Each structure factor is defined as follows:

$$F = \sum_j c_j f_j(Q) exp\left(iQz_j\right) exp[-\left(Qu_j\right)^2/2]$$

$f_j(Q)$ is the atomic scattering factor summed over all atoms for the respective substructure component. c_j, z_j and u_j are the occupancy, distance from the calcite surface and root mean square distribution width of atom j, respectively.

The analysis procedure was adapted from Fenter et al[3-5]. The unit cell form factor, F_{UC}, is calculated based on atom positions calculated from XRD data and is multiplied by the CTR form factor, $F_{CTR} = 1/[1 - exp(-iQc_{lat}/4)]$, where c_{lat} is the vertical calcite (104) lattice spacing (3.036 Å). The interfacial structure contains all atoms within the topmost six monolayers of the surface which were allowed to relax around their ideal lattice positions to yield best-fit results. Also, adsorbed species described by Gaussian distributions with the three parameters occupancy c, distance from the calcite surface z, and distribution width u were taken into account. The distribution of water above the actual sorption structure is described by a layered-water model. This model consists of a series of equally-spaced Gaussian peaks with the average electron density of bulk water (0.33 $e^-/Å^3$).

Several models applying varying starting conditions were tested to validate the reliability of the best-fit model. The least-squares fitting procedure yields the statistical uncertainty for each fitting parameter. Each fitting result is evaluated by a χ^2 value according to:

$$\chi^2 = \frac{1}{N_P}\sum_i \left(\frac{R_i - R_{calc}}{\sigma_i}\right)^2$$

where N_p is the number of data points, R_i and σ_i are the reflectivity and the uncertainty of the ith data point, respectively, and R_{calc} is the reflectivity calculated for the particular set of parameters from the model. Surface roughness is accommodated via incorporation of a roughness structure factor, whose behaviour is controlled by a parameter, β, related to the rms surface interfacial roughness as $= c_{lat}\sqrt{\frac{\beta}{1-\beta}}$. The angle-dependent absorption of X-ray photons by the solution film and the kapton containment foil is accounted for by a parameter in the fitting procedure.

**Lattice parameters**	**Best-Fit**		**Std Dev**
β (Robinson roughness)	0.23*		
Δ ($Ca^{\#1}$)	0.756	±	0.015
Δ ($Ca^{\#2}$)	1.088	±	0.020
Δ ($Ca^{\#3}$)	0.964	±	0.061
Δ ($Ca^{\#4}$)	0.028	±	0.005
Δ ($CO_3^{\#1}$)	0.958	±	0.021
Δ ($CO_3^{\#2}$)	0.063	±	0.017
Δ ($CO_3^{\#3}$)	0.880	±	0.042
Δ ($CO_3^{\#4}$)	1.102	±	0.044
$\theta^{\#1}$	36.3	±	1.1
$\theta^{\#2}$	44.9	±	1.5
$\theta^{\#3}$	39.4	±	1.1
$\theta^{\#4}$	46.6	±	1.1
$\phi^{\#1}$	2.9	±	14.4
$\phi^{\#2}$	-15.4*		
$\phi^{\#3}$	-8.8	±	2.7
$\phi^{\#4}$	6.7	±	7.3
**Fit quality**			
χ^2	1.35		
R-factor	0.075		

Table S1. Parameters of the CTR Best-Fit procedure (* fixed value)

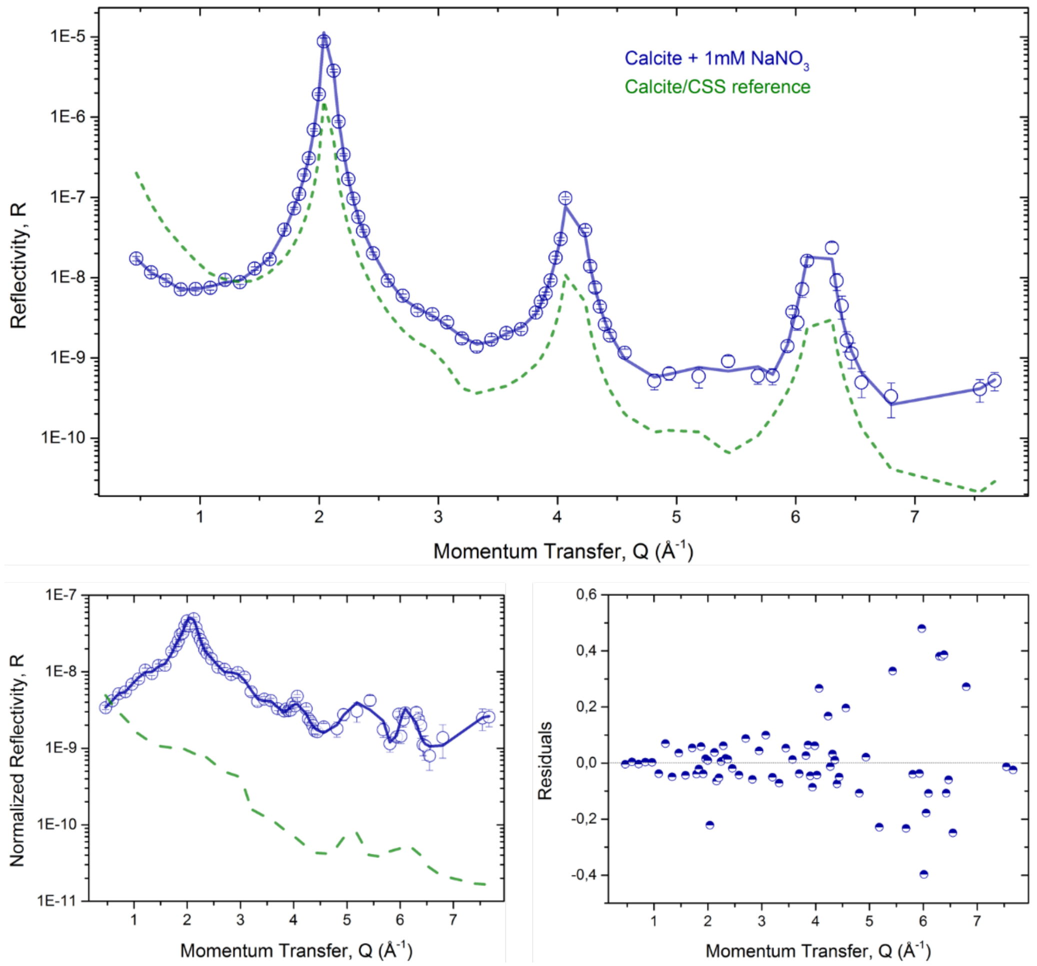

FIG. S 3. Left: Crystal truncation rod (CTR) of the calcite (104) surface in contact with 1 mM $NaNO_3$ for 2 weeks (blue circular data points) and respective best-fit model for this CTR (blue line). Calcite in contact with pure CSS is shown in green as a comparison (scaled, calculated from the literature[4]: the data is multiplied by a factor 10). Differences from the literature reference system are obvious. Especially the mid-zone (i.e. between the Bragg peaks at Q = 1.0, 3.1, 5.2 and 7.2) reflectivity which are highly sensitive to interfacial characteristics exhibit drastic changes. Normalized data and fit curve shown in the bottom left image, emphasizing the differences between the pristine calcite surface and the surface in contact with $NaNO_3$. Residuals of the fit displayed in the bottom right part.

Additional data and discussion

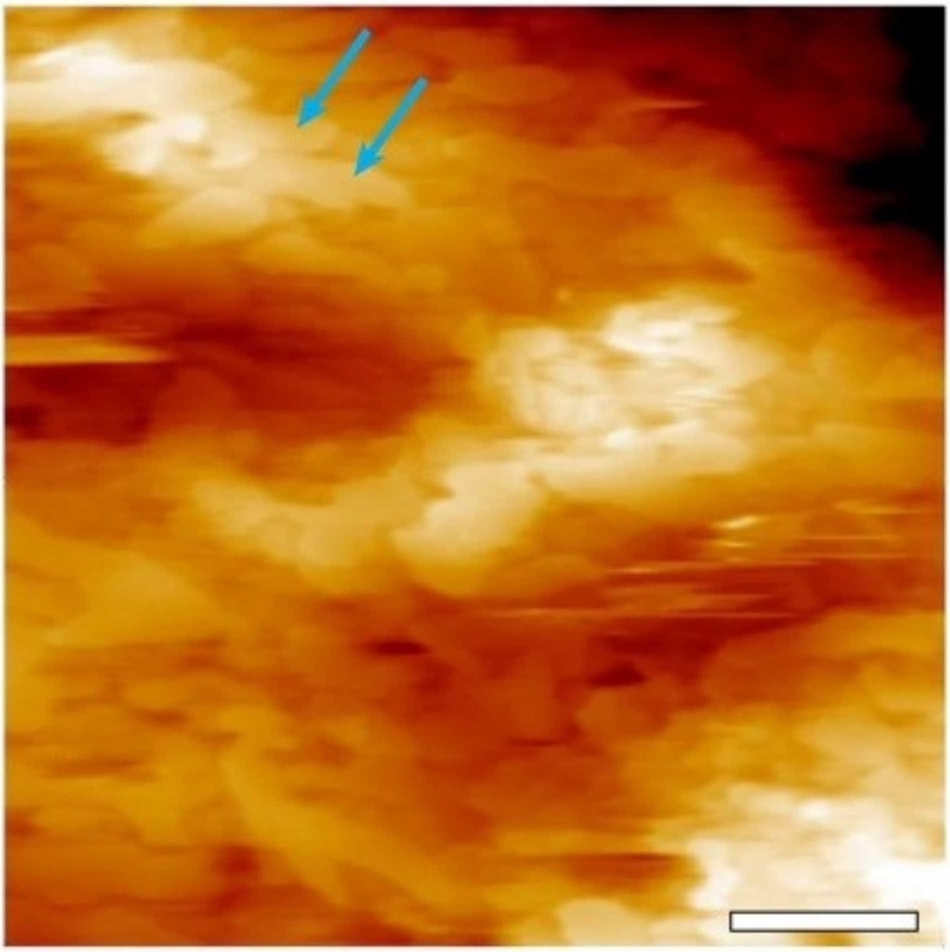

Fig. S 4. Calcite exposed to 1 mM KNO_3 solution for several days. The surface is dramatically restructured with many platelet-like features (arrow). The scale bar is 200 nm and the maximum height difference is 150 nm. KNO_3 was studied in order evaluate dependencies of the surface-restructuring from the cation. Differences between the $NaNO_3$ and KNO_3 system were observed. Here, the surface is not covered by an amorphous film, but divided into single, hard plates of crystalline material. Since K^+ is substantially larger than Na^+, this difference might be caused by the stronger affinity of Na^+ to the calcite surface.

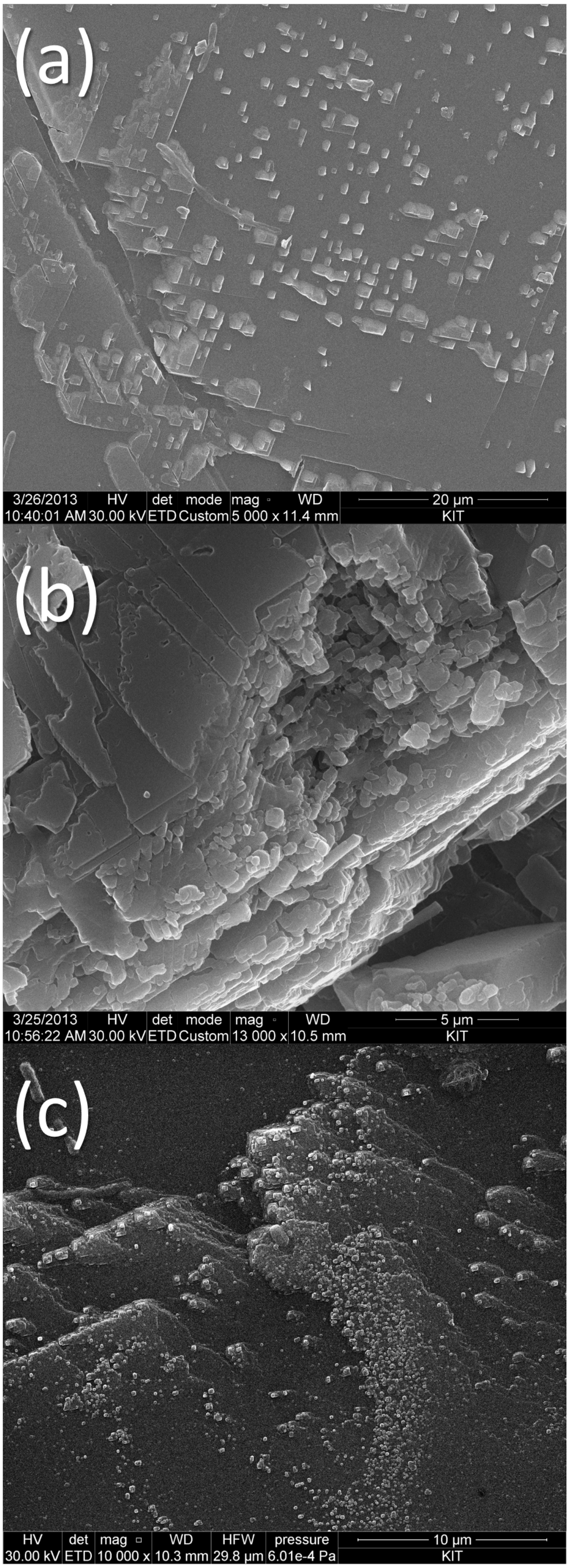
(a)
3/26/2013 10:40:01 AM
HV 30.00 kV
det ETD
mode Custom
mag 5 000 x
WD 11.4 mm
20 µm
KIT
(b)
3/25/2013 10:56:22 AM
HV 30.00 kV
det ETD
mode Custom
mag 13 000 x
WD 10.5 mm
5 µm
KIT
(c)
HV 30.00 kV
det ETD
mag 10 000 x
WD 10.3 mm
HFW 29.8 µm
pressure 6.01e-4 Pa
10 µm
KIT

Fig. S 5. Scanning electron micrographs of calcite (104) cleavage surfaces at 50 µm x 50 µm resolution. (a) Surface after one day contact with ultrapure water and rapid drying by dry N_2 stream. Clearly visible rhombohedral etch pits on the planes. (b) Calcite (104) exposed to CSS and additional 1 mM $NaNO_3$ for 24 hours. Drying procedure as above. Partial dissolution most prominently at step edges in agreement with our earlier study[6]. (c) Calcite after contact with 1 mM KNO_3 and identical drying procedure. Etch pits on the planes with size varying from nm- to µm scale and partially dissolved edges observable. The pits do not exhibit the clear rhombohedral shape as seen with ultrapure water in (a). Strong roughening of the surface and platelet-like features are in good agreement with AFM results.

Note on the surface charge of calcite

Measurements conducted on ultrapure calcite powder (MERCK, 0.7 m^2/g, 0.5g/l) showed the surface charge of the mineral to be negative at equilibrium (pH 8.3). We could not observe any measurable effect of the electrolyte solution used or its concentration on the measured surface charge which is also supported by earlier studies[7]. These results suggest the singe crystals used in this study to be always weakly negatively charged. The AFM tip conducting the measurement should be close to neutral at pH 8.3 if we assume that it is made primarily of silicon oxide[8].

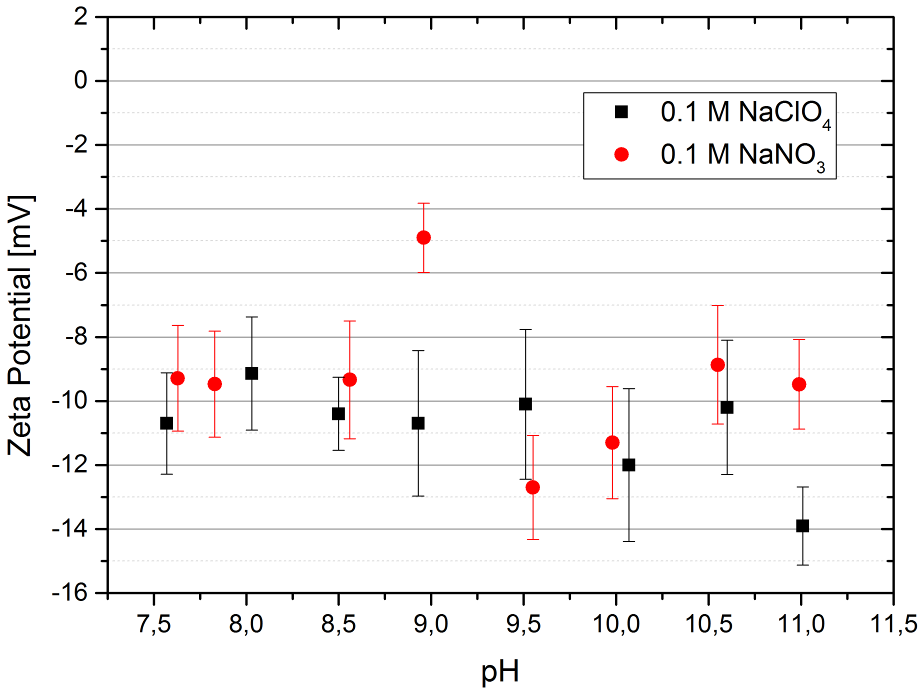

Fig. S 6. Zeta potential measurements of MERCK suprapure calcite powder (specific surface area 0.7 m^2/g) measured at solid to liquid ratio of 0.5 g/l in deionised water with 0.1 mol/l electrolyte. Measurement was performed on a Zetasizer Nano ZS by Malvern Instruments.

References

1. Fukuma, T., Ueda, Y., Yoshioka, S. & Asakawa, H. Atomic-Scale Distribution of Water Molecules at the Mica-Water Interface Visualized by Three-Dimensional Scanning Force Microscopy. *Phys Rev Lett* **104**, 016101 (2010).

2. Loh, S.H. & Jarvis, S.P. Visualization of Ion Distribution at the Mica-Electrolyte Interface. *Langmuir* **26,** 9176-9178 (2010).

3. Fenter, P. X-ray reflectivity as a probe of mineral-fluid interfaces: A user guide. *Rev Mineral Geochem* **49**, 149-220 (2002).

4. Fenter, P. & Sturchio N.C. Calcite (104)-water interface structure, revisited. *Geochim Cosmochim Ac* **97**, 58-69 (2012).

5. Fenter, P. & Sturchio, N.C. Mineral-water interfacial structures revealed by synchrotron X-ray scattering. *Prog Surf Sci* **77,** 171-258 (2004).

6. Hofmann, S., Voitchovsky, K., Schmidt, M. & Stumpf, T. Trace concentration - Huge impact: Nitrate in the calcite/Eu(III) system. Geochim Cosmochim Acta **125**, 528-538 (2014).

7. F. Heberling et al. Reactivity of the calcite–water-interface, from molecular scale processes to geochemical engineering. *Appl Geochem*, **45**, 158–190 (2014)

8. Zhao, C., Ebeling, D., Siretanu, I., van den Ende, D., & Mugele, F. Extracting local surface charges and charge regulation behavior from atomic force microscopy measurements at heterogeneous solid-electrolyte interfaces. Nanoscale **7,** 16298-16311 (2015).
